# In Utero Heat Stress Has Minimal Impacts on Processed Pork Products: A Comparative Study

**DOI:** 10.3390/foods11091222

**Published:** 2022-04-24

**Authors:** Siwen Xue, Jun-young Park, Jacob R. Tuell, Jacob M. Maskal, Jay S. Johnson, Thu Dinh, Yuan H. Brad Kim

**Affiliations:** 1 Meat Science and Muscle Biology Laboratory, Department of Animal Sciences, Purdue University, West Lafayette, IN 47907, USA; vic_xsw0911@163.com (S.X.); skyg5252@gmail.com (J.-y.P.); tuell@purdue.edu (J.R.T.); jmaskal@purdue.edu (J.M.M.); 2 Key Laboratory of Meat Products Processing, Ministry of Agriculture, Jiangsu Collaborative Innovation Center of Meat Production and Processing, Quality and Safety Control, College of Food Science and Technology, Nanjing Agricultural University, Nanjing 210095, China; 3 USDA-ARS Livestock Behavior Research Unit, West Lafayette, IN 47907, USA; jay.johnson2@usda.gov; 4 Department of Animal and Dairy Sciences, Mississippi State University, Starkville, MS 39762, USA; thu.dinh@msstate.edu

**Keywords:** gestational heat stress, pork patty, pork sausage emulsion, quality attributes, fatty acid composition

## Abstract

This study aimed to determine what effects in utero heat stress (IUHS) in pigs may have on quality of processed pork products. In two experiments, patties and emulsion sausages were prepared from lean and fat from pigs subjected to IUHS or in utero thermoneutral (IUTN) conditions. Patties formulated to contain 25% added fat had altered textural properties compared to those without additional fat, as shown by lower hardness, cohesiveness, springiness, and chewiness values (*p* < 0.05), which was not affected by IUHS treatment. Neither fat content nor IUHS treatment affected fluid losses of patties (*p* > 0.05). In general, 25% added fat patties had greater L*, a*, b*, hue angle, and chroma values than lean patties (*p* < 0.05). However, 25% added fat patties from the IUHS treatment maintained superior color stability during aerobic display, despite lean patties from this treatment exhibiting increased lipid oxidation (*p* < 0.05). For emulsion sausages, minimal differences in quality attributes and oxidative stability were found between treatment groups. Subcutaneous fat from IUHS pigs had greater C20:1 and C20:2 than IUTN (*p* < 0.05), although the magnitude of these differences was slight. Overall, the findings of this study suggest IUHS would have minimal impacts on the functional properties of raw pork, resulting in similar final quality of processed products to IUTN.

## 1. Introduction

Heat stress (HS) in swine production impairs animal welfare and production efficiency [1]. Although cooling systems have been utilized to combat HS, the detrimental effects are unavoidable to some extent during the summer season [2]. At present, the effects of acute and chronic postnatal HS on physiology, performance, and quality of pork products have been well studied [3]; however, significant gaps in relation to prenatal HS on these attributes, especially meat quality, remain unclear. It has been demonstrated that in utero HS (IUHS) may lead to changes to metabolic traits, including insulin and glucose regulation, thereby affecting animal performance [4,5]. As a result, pork carcasses from pigs subjected to IUHS conditions may have altered composition as the accretion of lipid at the expense of lean tissue is favored [5,6,7,8]. Recently, our parallel study found that the objective tenderness of porcine longissimus lumborum muscles subjected to IUHS would be poorer than in utero thermoneutral (IUTN) counterparts [9]. However, at present, the implications to quality of processed pork products remain largely unknown.

Fat, especially as relates to fatty acid composition, has a key role in determining the quality attributes of processed pork products. The composition of fatty acids is known to be affected by numerous live animal factors, including genetics, nutrition, and physiology [10]. Pork products with a greater concentration of long chain polyunsaturated fatty acids are known to have inferior oxidative stability during storage, as well as decreased firmness, which would, in turn, affect processability and quality attributes [10]. Johnson et al. reported that the lipid accretion rates of IUHS pigs during the finishing phase were greater than IUTN counterparts, regardless of postnatal environmental conditions. At present, the implications to fatty acid profile are poorly categorized. Considering the widespread use of porcine subcutaneous fat in the manufacture of processed pork products, determining if IUHS would influence quality attributes would be beneficial to the industry. Therefore, the main objective of this study was to evaluate and compare the quality attributes of two common pork products (patties and emulsion sausages) using lean and fat from IUHS and IUTN pigs.

## 2. Materials and Methods

### 2.1. Animal Care and Use

The University of Missouri Animal Care and Use Committee approved all procedures involving the dams subjected to HS or thermoneutral (TN) conditions (#9340). Approval from the Purdue Animal Care and Use Committee was gained for all procedures relating to the transported offspring (#1806001756). Care of offspring was in adherence to the *Guide for the Care and Use of Agricultural Animals in Research and Teaching* [11].

Detailed information regarding the treatments can be found in the publications by Maskal et al. [12] and Tuell et al. [9]. In brief, dams (*n* = 24) were blocked by body weight and distributed among treatments (*n* = 12/treatment). Of the 24 total dams that were bred, 9 from the TN group became pregnant and 12 from the HS group became pregnant. Dams were first generation Landrace × Large White gilts, and sires were a single Duroc line (Choice Genetics, USA, Des Moines, IA, USA). All dams were kept in TN conditions during the first 5 d following insemination. The treatment group consisted of cyclical HS, in which the conditions of the chamber during the daytime were 35.8 ± 0.2 °C and night time were 28.4 ± 0.2 °C. Relative humidity of the HS chamber was regulated at 80.9 ± 6.0%. Prior to reaching these conditions at d 11, an acclimation period from d 6 to d 10 was allowed. The TN conditions were kept constant at 17.5 ± 2.1 °C at 70.2 ± 8.8% relative humidity. At d 59 following insemination, all dams were kept in similar TN conditions until farrowing. Throughout the study, dams were provided water ad libitum. During gestation, dams were limit fed with 2.0 kg/d of a corn and soybean-based diets to meet or exceed nutrient requirements [13], detailed by Maskal et al. [12]. After farrowing, feed was provided ad libitum. Weaned offspring were transported approximately 11 h and 40 min from Columbia, MO, USA, to West Lafayette, IN, USA. Afterwards, offspring were raised in similar conditions, housed in 40 mixed sex pens with 4 individuals per pen (*n* = 160). At harvest, 10 gilts per treatment group (*n* = 10 IUTN and 10 IUHS) were selected and blocked by body weight (117.3 ± 1.7 kg). Detailed information regarding the harvest conditions can be found in Tuell et al. [9]. At 7 d postmortem, legs and subcutaneous backfat were collected from the right side of each carcass and processed as later described.

### 2.2. Sample Preparation

Patties and emulsion sausages were manufactured using lean and fat from IUHS and IUTN pigs. Experiments were designed to evaluate the quality attributes of products according to how they are typically marketed in retail settings, where patties are sold raw and emulsion sausages are sold as fully cooked, ready-to-eat products.

#### 2.2.1. Manufacturing of Patties

Lean muscle was separated from pork legs, removing visible fat and connective tissue. Fat was collected from the dorsal region of the carcass with skin removed. Both lean and fat were cut into 1.5 cm cubes prior to grinding separately through a 0.25 in plate (M-12-FS, Torrey, Monterrey, NL, Mexico). In a factorial arrangement, preparations consisting of lean and fat were made: 100% IUHS lean (HS_0), 75% IUHS lean + 25% IUHS fat (HS_25), 100% IUTN lean (TN_0) and 75% + 25% TN fat (TN_25). All treatments were mixed by hand for 90 sec prior to manufacturing of patties. A total of 11 patties, each weighing approximately 100 g, was prepared per batch, and three replications were conducted. Three patties were overwrap packaged with polyvinylchloride film (oxygen transmission rate of 23,000 cm^3^ O_2_/m^2^/24 h at 23 °C) and displayed under fluorescent lighting (3500 lx) for 4 d at 2 °C. Instrumental color attributes of patties were evaluated daily, and samples were also collected at 0 d and 4 d for determinations of display loss and lipid oxidation. Four patties were used for cook loss and textural properties on the processing day. The remaining four patties per treatment were vacuum packaged, immediately frozen at −40 °C, and stored for 21 d before determination of freeze/thaw loss.

#### 2.2.2. Manufacturing of Emulsion-Type Sausages

Emulsion sausages were prepared based on a previous protocol [14] with slight modifications. Preparations were comprised of 50% lean, 30% fat, 17.5% ice water, 2% NaCl, 0.3% sodium triphosphate salt, and 0.012% sodium nitrite. A 2 × 2 factorial design was used to manufacture four different combination treatments of lean (L) and fat (F): IUHS lean and IUHS fat (HSL–HSF), IUHS lean and IUTN fat (HSL–TNF), IUTN lean and IUHS fat (TNL–HSF), IUTN lean and IUTN fat (TNL–TNF). Emulsions were manufactured by using a bowl cutter (Cutter C4, Sirman, Marsango, Italy), where weighed ice water was added to the emulsion during chopping to minimize the temperature rise (<7 °C). Afterwards, raw emulsions were added into 50 mL centrifugal tubes (60 g per tube). Tubes were held at 2 °C overnight before cooking in a water bath for 30 min at 70 °C. Cooked sausages were removed from the tubes, and extruded fluids were collected from at least 3 samples for the determination of fat-binding capacity. All the cooked emulsions were vacuum packaged and stored at 2 °C in dark conditions for 0, 3, 6, 9, and 18 d for the following measurements of proximate compositions, textural profiles, pH values and lipid oxidation.

### 2.3. Proximate Composition

Moisture content and ash content of patties and emulsion sausages were performed following AOAC Official Method 950.46(b) and AOAC Official Method 920.15, respectively [15]. Protein content was determined using the Laboratory Equipment Corporation (LECO) nitrogen analyzer using ethylenediamine tetraacetic acid (EDTA) as standard (Leco Corp., St. Joseph, MI, USA). Values are presented on a wet matter basis (%). All the measurements were performed in triplicate (*n* = 3).

### 2.4. Moisture Loss of Pork Patties

Moisture loss was determined as an indirect indication of water-holding capacity by measuring multiple weight losses during further processing [16]. Display loss was measured by weighing patties individually before and after the 4 d display period. Surface moisture was blotted using paper towel before weighing the samples, and display loss was expressed as the percent weight difference between patty weights at 0 and 4 d.

Cook loss of patties was expressed as the weight differences before and after cooking, expressed on a percentage basis. Patties were cooked on an electronic grill until the core temperature reached 72 °C, which was monitored by using a T type thermocouple (Omega Engineering, Stamford, CT, USA) connected to an OctTemp 2000 data logger (Madge Tech, Inc., Warner, NH, USA). Cooked patties were stored overnight at 4 °C before recording the cooked weight, wrapped with aluminum foil to prevent evaporative moisture loss. Samples for cook loss were subsequently used for textural profile analysis.

Freeze/thaw loss of pork patties was determined after 21 d of frozen storage by comparing the weight difference between fresh and thawed patties. Weight of pork patties were recorded on the manufacturing day. To determine the thawed weight, patties were transferred from freezer to the 4 °C walk-in cooler, sitting in the cooler overnight till fully thawed. Followed by removing patties from the package, fluid on patties were blotted gently using paper towel before recording the weight (*n* = 4).

### 2.5. Cook Loss and Fat-Binding Capacity of Emulsion-Type Sausages

Briefly, the cook loss of samples was determined by collecting the weight of emulsions before and after cooking as previously described (70 °C for 30 min in a water bath). Cook loss was expressed as a percentage of the released fluid relative to the initial sample weight. These samples were also used for the textural analysis afterwards.

Fat-binding capacity was determined as the released fluid weight remained after heating (16 h/105 °C in a drying oven) and expressed as percentage of initial sample weight, the method of which was modified from the one used before [17]. A higher value indicates an inferior fat-binding capacity of emulsion. Three determinations for each sample were carried out during storage for each time point.

### 2.6. Textural Profile Analysis

Textural profile analysis (TPA) of patties and emulsion sausage were determined using a TA-XT Plus texture analyzer (Stable Micro System Corp., Surrey, UK) by following the previous protocol [18]. Both pork patties and emulsion sausage were subject to texture analysis using the same samples from cooking loss. For pork patties, three cores from one patty were moved and subjected to the textural analyzer, and a total of 4 patties were used for each batch of trial. Three fractions of one sausage emulsion link were cut into cylinders with 22 mm diameter and 20 mm height. At least three sausage emulsion link were used for each independent trial.

Samples were compressed twice to 50% of their original height with a compression plate of 50 mm diameter. The deformation curves of force-distance were recorded at a trigger force of 5 g, a crosshead speed of 5 mm/s, and a recording speed of 5 mm/s. Hardness (N), adhesiveness, cohesiveness, gumminess, chewiness (N), and resilience values were obtained and analyzed. A minimum of 3 replicates per batch was conducted.

### 2.7. pH Measurement

The pH was determined over the storage period in quadruplicate by a pH-meter (Sartorius Basic Meter PB-11 Sartorius AG, Göttingen, Germany) at room temperature on homogenates of 1.0 g of sample and 10 mL of distilled water.

### 2.8. Lipid Oxidation

According to the protocol described by Kim et al. [19] approximately 5.0 g of raw patty or cooked emulsion sausage was homogenized with 15 mL of distilled water together with 50 μL of 10% (*v*/*v*) butylated hydroxy anisole solution, which was solubilized in 90% (*v*/*v*) of ethanol, at a speed of 9200 rpm (Ultra-Turrax T25, Janke & Kunel IKA-Labortechnik, Staufen, Germany). An aliquot of 1 mL of the homogenate was mixed with 2 mL of 20 mM thiobarbituric acid (TBA) solution. The mixture was incubated in a water bath at 80 °C for 15 min, followed by immediate cooling down in an ice bath for another 10 min. Afterwards, mixtures were centrifuged (2000× *g*, 10 min, 4 °C); the supernatants were filtered through a filter paper (Whatman No. 4). Absorbance reading was recorded at 531 nm (Epoch, Biotek Instruments Inc., Essex Junction, VT, USA). The thiobarbituric acid reactive substances (TBARS) value was presented as mg malondialdehyde (MDA)/kg sample and was calculated using molecular extinction coefficient of 1.56 × 10^5^ mol^−1^ cm^−1^.

### 2.9. Instrumental Color

The surface color and color stability of pork patties during display were determined using a HunterMiniScan EZ colorimeter (Hunter, Reston, VA, USA) with D65 illuminant with the aperture of 25 mm and observer angle of 10°. Calibration was conducted following standard instruction using white tile. CIE L* (lightness), CIE a* (redness), and CIE b* (yellowness) values of samples were collected after calibration. Hue angle and chroma value of samples were calculated according to the following formula based on the AMSA guideline [20]: hue angle = tan^−1^ (b*/a*) and chroma value = (a*2 + b*2)^1/2^.

### 2.10. Fatty Acid Profiling

#### 2.10.1. Methyl Ester Preparation

Approximately 100 mg of subcutaneous fat sample was weighed out and put into a 20 mL flat-bottom glass vial with a Teflon^®^-lined screw cap. Methylation of fatty acids (FAs) was performed using a direct trans-esterification method, as described by O’Fallon et al. [21]. Briefly, samples were first saponified in the presence of potassium hydroxide (KOH) and methanol. Saponified FAs were trans-esterified by adding sulfuric acid into the mixture. Fatty acid methyl esters (FAME) were extracted by hexane.

#### 2.10.2. Gas Chromatography-Mass Spectrometry (GC-MS) Determination

Fatty acid methyl esters (FAMEs) were subject to a GC-MS (gas chromatography-mass spectrometry) system (Agilent Technologies, Santa Clara, CA, USA) for quantification. The GC-MS system used was equipped with an HP-88 capillary column (30 m × 0.25 mm i.d. × 0.2 µm film thickness; Supelco Inc., Bellefonte, PA, USA) and an Agilent 5975C inert XL MSD with triple-axis mass detector. Peaks were identified by FAME standards in Supelco^®^ 37 Component FAME Mix (Sigma-Aldrich, St. Louis, MO, USA), FAME #21 Mix (AOCS #6; Restek, Bellefonte, PA, USA), a customized 17-component FAME mix (Nu-Chek-Prep, Elysian, MN, USA), and selected individual fatty acid standards. All FAs were monitored by their target qualifier ions in a selected ion monitoring mode. Quantification of FA concentration (µg/g of sample) was performed using an internal standard calibration method. The result is presented in FA percentage (g/100 g of total FAs).

### 2.11. Statistical Analysis

Both pork patty study and emulsion study were completely randomized block design, and a total of three independent batches was performed. For the pork patties, both fat content (0% vs. 25%) and treatment condition (IUHS vs. IUTN) were considered as the main factors. When the samples involve the display or storage, different storage/display times were considered as the main effects, and the interaction between treatment and storage/display time was analyzed. Regarding the pork emulsion study, treatment was considered as the main effect. FA results were analyzed under the *t*-test program as only treatment (IUHS vs. IUTN) was considered as the factor. All data were analyzed using the PROC MIXED procedure of SAS 9.4 software (SAS Institute Inc., Cary, NC, USA). The least square means for all traits were separated (F test) at the confidence level of 95% (*p* < 0.05).

## 3. Results and Discussion

### 3.1. Proximate Composition

Pork patties and emulsion-type sausages were manufactured using lean meat and subcutaneous fat collected from either IUHS or IUTN pork. Results of statistical analysis and proximate compositions of patty and emulsion are displayed in Table 1, Table 2 and Table 3. For pork patties, it was found that gestational condition had a significant impact on the fat content of patties, where patties made from IUHS lean and fat had a lower fat content compared to the IUTN patties (*p* < 0.05). As expected, patties with 25% subcutaneous fat had considerable differences in proximate composition (Table 1, *p* < 0.0001), as higher lipid content and lower protein and ash contents of TN_25 and HS_25 were observed. Regarding the pork sausage emulsion, as shown in Table 2, moisture content and fat content of cooked emulsions were both affected by lean meat type (*p* < 0.05) and lean–fat interaction (*p* < 0.05). A combination of HSF and HSL resulted in pork emulsions with the lowest moisture content yet the highest fat content compared to the other three combinations (*p* < 0.05). This could be attributed to the inferior water-binding capacity of emulsions made from HSF and HSL. The crude protein content of emulsions was significantly influenced by the fat type, as HSF-based emulsions have less protein compared to the TNF counterparts, regardless of lean type (Table 2). In short, fat content is a major factor that contributed to crude composition difference in patties, while for sausage emulsions, lean and fat sources did not bring practical changes in proximate composition.

### 3.2. Fluid Losses

Display loss (DL, %), freeze/thaw loss (F/T L, %), and cook loss (CL, %) are important parameters to evaluate the quality of meat patties [22]. As shown in Table 1, none of the studied factors (i.e., gestational condition, fat content, or their interactions) had influences on the water-holding capacity of patties (*p* > 0.05). Therefore, in agreement with a previous study, fat content did not have a significant influence on drip loss, cook loss, and expressible moisture of pork patties [23]. Moreover, the gestational condition does not play a role in affecting the water-holding capacity of pork myofibrillar protein in patties.

For sausage emulsion, lower cook loss and higher fat-binding capacity indicate superior quality [22]. As displayed in Table 2 and Table 4, the cooking loss of emulsions was significantly affected by the fat types, showing that emulsions made of HSF generally have lower cooking loss than the TNF–based emulsions (*p* < 0.05). However, the variation was only around 0.17%; therefore, no practical differences would be recognized in terms of industrial manufacturing yield. As for fat-bind capacity, there is no difference among treatments (*p* > 0.05). Since myofibrillar protein is the major fraction that exerts water-/fat-binding capacities [24,25], it is reasonable to postulate that IUHS has limited impacts on the functional properties of myofibrillar proteins. Additionally, the processing operations such as chopping, a strong external mechanical force, might play a role in outweighing the potential negative impacts of IUHS on the functional properties of myofibrillar protein [26].

### 3.3. Texture Profile Analysis

As displayed in Table 1, texture attributes of pork patties were not affected by gestational conditions (*p* > 0.05), while these characteristics were influenced by fat content (*p* < 0.05) except for adhesiveness (*p* > 0.05). Generally, patties with 25% added fat had significantly lower hardness, resilience, cohesiveness, springiness, gumminess, and chewiness compared to the patties comprised on lean only, regardless of gestational conditions. Given that fat is used for improving the texture attributes of meat products, it is understandable that pork patties with 25% added fat had lower hardness and gumminess values compared to the patties without extra fat addition. Moreover, the hardness of patties was significantly affected by the interaction between gestational condition and fat content (Table 1, *p* = 0.0318). As found, HS_0 patties maintained the highest hardness value among treatments (*p* < 0.05, Table 5), while patties made of either IUTN or IUHS meat were not different from each other (*p* > 0.05). Previously, Foxcroft et al. [27] found that muscle phenotype, as well as biological characteristics of piglets, can be greatly affected by the in utero conditions, possibly due to the critical step of embryonic myogenesis affected during gestation. This could potentially explain that the lean portion from the IUHS pork carcasses is tougher than the IUTN lean portion, as a higher shear force value of IUHS longissimus lumborum muscles compared to IUTN counterparts was observed in our parallel project [9]. However, as a processed product, the undesirable tough texture of HS_0 patties could be improved by including higher fat content regardless of gestational condition.

In contrast, for pork sausage emulsions, as presented in Table 2 and Table 4, no significant differences in TPA attributes were found among the four treatments in the current study (Table 2, *p* > 0.05), except for springiness, which was influenced by the interaction between fat and lean source (*p* < 0.05). This result, to some extent, verified that the functional properties of myofibrillar proteins are not significantly affected by IUHS treatment, in agreement with the results of fluid losses presented above.

### 3.4. pH Values

Alterations in pH values for both patty and emulsion during the simulated retail storage period were determined. For pork patties, as shown in Table 1, during 5 days of storage, none of the factors (i.e., gestational condition, fat content, storage, and their two-way or three-way interactions) were found to have significant influences on pH value (*p* > 0.05). This result is in line with the findings from Tuell et al. [9], where no significant differences in pH between IUHS and IUTN loin muscles were determined throughout the 7 days of aging [9].

In contrast, pH value changes in pork sausage emulsions during 18 days of storage were influenced by the storage period and the fat–lean interaction (*p* < 0.05, Table 2). Generally, HSL–HSF showed a pH value of 5.68, which is lower than the other three treatments that were not different from each other (*p* > 0.05, Table 5). Earlier, several studies reported that the pH value was higher in the cooked emulsion batter than the raw batter, potentially due to the release of imidazolium from the basic R group of histidine [28,29]. We, therefore, assumed that the emulsion made of IUHS lean and IUHS fat might have a lower level of imidazolium release during cooking compared to the other three treatments. Previous studies confirmed that exposure to heat stress when the pigs were in utero could alter several physiological and metabolic cues of swine, such as energy metabolism, glucose metabolism, etc., compared to the IUTN pigs [30]. However, the exact mechanism needs to be investigated in the future.

Although the pH value was affected by the IUHS and IUTN meat/fat combination, no significant influence of fat–lean interaction on cooking loss or fat-binding capacity was observed (*p* > 0.05, Table 2). Therefore, the divergence in pH values among treatments throughout the storage would unlikely affect the fluid holding capacity of pork emulsions.

### 3.5. Lipid Oxidation

Lipid oxidation is considered the major cause of quality deterioration in meat products, resulting in unfavorable color, flavor, texture, etc. [31]. As shown in Table 1, the storage period is the sole factor that had a significant impact on the TBARS value of pork. The TBARS value for patties increased as time extended from d 0 to d 4 as oxidation products tend to accumulate during aerobic storage. No significant interactions of gestational condition, fat content, and storage period were found for the current study (*p* > 0.05).

Regarding pork sausage emulsions, none of the factors has an impact on the TBARS value of the samples (Table 2, *p* > 0.05), indicating that gestational heat stress did not affect the lipid oxidative capacity of pork. Therefore, it is concluded that lean and fat collected from the IUHS pigs had negligible influence on the oxidative stability of either pork patties or pork sausage emulsions.

### 3.6. Instrumental Color

As an important quality characteristic of patty products, color attributes throughout the retail period are decisive factors that affect the purchase decision of consumers [32]. As displayed in Table 1, redness, hue angle, and chroma values are influenced by the gestational condition (*p* < 0.05). In addition to the fat content, which showed significant impacts on all the color attributes of pork patties, the storage period affected the color attributes of pork patties (*p* < 0.05), although the hue angle was close to statistical significance (Table 1, *p* = 0.0501). Moreover, the interaction between gestational condition and fat content exerted significant influences on L*, b*, hue angle, and chroma values (*p* < 0.05). The effect of interaction on color attributes is exhibited in Table 6, showing the results of lightness (L*), yellowness (b*), hue angle, and chroma, respectively. As shown in Table 6, pork patties that included 25% of fat generally maintained a higher value of lightness, which is likely attributed to the additional fat inclusion. According to Bhattacharya et al., higher fat content in the product can reflect more surface light, resulting in higher lightness [33]. Yellowness followed a similar trend, with HS_25 and TN_25 patties maintaining higher a* and b* values than the patties without additional fat throughout the display period. The CIE a* value is primarily attributed to the content and/or status of myoglobin [34]. Given the complexity of the mechanism underlying lipid and myoglobin oxidation interaction, it is assumed that in the present study condition, lipid oxidation may act as a facilitator of myoglobin oxidation, manifested as slowed myoglobin oxidation [35]. As a result, the process of discoloration of high-fat patties was delayed. Although the discoloration extent of HS_25 and TN_25 patties was greater than the other two treatments, as shown by higher hue angle values throughout the display period, the chroma values of HS_25 and TN_25 were also higher than the HS_0 and TN_0 patties (*p* < 0.05). Considering the overall change in color attributes during storage, the HS_0 patties maintained a higher redness and higher color saturation compared to the other three treatments. Therefore, beyond our expectations, lean and fat collected from the IUHS pig may exert some positive impact on the color attributes of the pork patty.

### 3.7. Fatty Acid Profiling

The composition of fatty acids in animal adipose tissue can be largely affected by diets and growth performance [10]. As previous studies reported, IUHS can greatly change the growth performance of piglets, leading to variations in energy metabolism [6]. However, it is unclear if the fatty acid compositions, which have a great influence on the quality of meat products, would be affected by these changes or not. It is known that different ratios of polyunsaturated FAs (PUFAs), monounsaturated FAs (MUFAs), and saturated FAs may affect the oxidative stability of meat products to a certain extent [36]. Generally, higher content of unsaturated FAs denotes a higher susceptibility to oxidation [10]. Therefore, the FA composition of adipose tissues is highly related to the quality attributes of manufactured meat products. The FA compositions of IUHS and IUTN subcutaneous fat are displayed in Table 7. Although it was expected that the FA profiles between IUHS and IUTN subcutaneous fat would be significantly different, most FAs were not different between treatments aside from C20:1 cis 11 and C20:3 cis5 cis8 cis11 (Table 7, *p* < 0.05). Although studies found that a slight difference in C18:3n-3 (1.3% vs. 0.85%) in bacon led to an increased TBARS value after 7 days of retail display [37], no significant difference in total PUFAs in IUHS and IUTN subcutaneous fat was found. In addition, the dominant MUFAs and PUFAs, such as C18:1 cis9 (30.22% vs. 30.07%) and C18:2 cis9 cis12 (19.32% vs. 18.96%), were not different between IUHS and IUTN fats. Therefore, the inclusion of subcutaneous fat from IUHS would be unlikely to influence the quality, especially the oxidative stability, of processed meat products. The obtained results of FA composition denoted that IUHS has limited impacts on the FA composition of subcutaneous fat; moreover, FA profiles could explain the TBARS result in part, as no significant difference was found between samples manufactured using IUHS fat or IUTN fat.

## 4. Conclusions

In general, the findings of this study suggest that lean and fat from pork subjected to IUHS would not be considerably different from the meat quality and oxidative stability of IUTN counterparts. For pork patties, most measures were affected by fat content only rather than IUHS treatment; however, there was some evidence to suggest HS_25 patties would exhibit equivalent or superior color and color stability compared to other treatment groups. Minimal differences between treatments were found among properties of emulsion sausages, suggesting meat proteins maintain normal functionality. Similarly, the fatty acid composition of subcutaneous backfat was largely unaffected by the in utero heat stress, which could explain why the products maintained comparable oxidative stabilities.

## Figures and Tables

**Table 1:** Main and interactive effects of gestational condition, fat content, and storage period on quality attributes of pork patties.

		IUC		FC		Storage (S)		IUC*FC		IUC*S		FC*S		IUC*FC*S	
		SEM	*p* Value	SEM	*p* Value	SEM	*p* Value	SEM	*p* Value	SEM	*p* Value	SEM	*p* Value	SEM	*p* Value
Proximate	Moisture	0.4024	0.1556	0.4024	<0.0001	/	/	0.5691	0.3296	/	/	/	/	/	/
	Protein	0.1926	0.2398	0.1926	<0.0001	/	/	0.2554	0.6578	/	/	/	/	/	/
	Ash	0.0260	0.6250	0.0260	<0.0001	/	/	0.0367	0.9633	/	/	/	/	/	/
	Fat	0.5001	0.0439	0.5001	<0.0001	/	/	0.7072	0.3592	/	/	/	/	/	/
Fluid Loss	Drip loss	1.167	0.1601	1.167	0.3142	/	/	1.650	0.5759	/	/	/	/	/	/
	F/T loss	2.063	0.6299	2.063	0.0555	/	/	2.238	0.7878	/	/	/	/	/	/
	Cooking loss	1.765	0.6169	1.765	0.7556	/	/	2.495	0.4132	/	/	/	/	/	/
Textural profile analysis	Hardness	789.4	0.4172	789.4	<0.0001	/	/	1001.4	0.0318	/	/	/	/	/	/
	Adhesiveness	0.1147	0.555	0.1147	0.1655	/	/	0.1498	0.2191	/	/	/	/	/	/
	Resilience	0.9493	0.4551	0.9493	<0.0001	/	/	1.3425	0.329	/	/	/	/	/	/
	Cohesiveness	0.0183	0.6496	0.0183	<0.0001	/	/	0.0259	0.6611	/	/	/	/	/	/
	Springiness	2.322	0.7332	2.322	0.001	/	/	3.284	0.4016	/	/	/	/	/	/
	Gumminess	546.8	0.2689	546.8	<0.0001	/	/	739.4	0.0545	/	/	/	/	/	/
	Chewiness	467.3	0.2585	467.3	<0.0001	/	/	644.0	0.0555	/	/	/	/	/	/
Physicochemical property	pH values	0.0420	0.2355	0.0420	0.7074	0.0420	0.2646	0.0519	0.9420	0.0519	0.8719	0.0519	0.9722	0.0675	0.9722
	TBARS	0.0977	0.6421	0.0977	0.8603	0.0989	0.0063	0.1002	0.5864	0.1027	0.3427	0.1027	0.2015	0.0782	0.2707
Color attributes	L* value	0.5177	0.0633	0.5177	<0.0001	0.6040	0.0002	0.5767	0.0005	0.7254	0.9405	0.7254	0.0588	0.9213	0.9828
	a* value	0.6081	0.0204	0.6081	<0.0001	0.8295	0.0387	0.7628	0.1487	1.104	0.5994	1.104	0.5179	1.510	0.6141
	b* value	0.6305	0.6742	0.6305	<0.0001	0.6551	0.0073	0.647	<0.0001	0.6942	0.4530	0.6942	0.0912	0.7665	0.9781
	Hue	1.524	0.0021	1.524	<0.0001	1.672	0.0501	1.6246	0.0003	1.894	0.9663	1.894	0.5020	2.272	0.8368
	Chroma	0.8178	0.0262	0.8178	<0.0001	0.9795	0.0259	0.9287	0.0325	1.202	0.4538	1.202	0.3112	1.553	0.6038

Note: IUC—In utero Condition; FC—Fat Content; Storage—S; SEM—Standard error of means; Data from three independent trials were analyzed using the PROC MIXED procedure. IUC*FC: interaction between IUC and FC; IUC*S: interaction between IUC and S; FC*S: interaction between FC and S; IUC*FC*S: three way interaction between IUC, FC and S; Main effect of in utero conditions, fat content, storage period as well as their interactions on the tested parameters were evaluated. SEM and *p* values were presented; Main factor with a *p* value < 0.05 is considered as having significant impact on the measured attributes, and detailed comparison will be performed and listed in the corresponding tables.

**Table 2:** Main and interactive effects of fat source, lean source, and storage period on quality attributes of emulsion sausages.

		Fat		Lean		Storage (S)		Fat*Lean		Fat*S		Lean*S		Fat*Lean*S	
		SEM	*p* Value	SEM	*p* Value	SEM	*p* Value	SEM	*p* Value	SEM	*p* Value	SEM	*p* Value	SEM	*p* Value
Proximate	Moisture	0.5186	0.8555	0.5186	0.0332	/	/	0.3667	0.0052	/	/	/	/	/	/
	Protein	0.1554	0.0271	0.1554	0.2765	/	/	0.2198	0.2005	/	/	/	/	/	/
	Ash	0.0460	0.8600	0.0460	0.0721	/	/	0.0651	0.9186	/	/	/	/	/	/
	Fat	0.3881	0.1995	0.3881	0.0034	/	/	0.3659	0.0010	/	/	/	/	/	/
Functional property	Cooking loss	0.0856	0.0426	0.0856	0.3377	/	/	0.1106	0.7347	/	/	/	/	/	/
	Fat-binding	0.7525	0.6535	0.7525	0.5246	/	/	0.8337	0.8942	/	/	/	/	/	/
	pH	0.0812	0.1597	0.0812	0.1735	0.0559	0.0446	0.0623	0.0057	0.0559	0.4843	0.0559	0.8168	0.0803	0.8223
Lipid oxidation	TBARS	0.0213	0.4145	0.0213	0.3469	0.0213	0.8112	0.0200	0.6596	0.0200	0.1810	0.0200	0.1991	0.0313	0.3614
TPA	Hardness	373.4	0.1544	373.4	0.4330	/	/	262.84	0.0794	/	/	/	/	/	/
	Adhesiveness	2.5667	0.6949	2.5667	0.3708	/	/	1.9804	0.1459	/	/	/	/	/	/
	Resilience	1.8300	0.8171	1.8300	0.4988	/	/	1.6619	0.1420	/	/	/	/	/	/
	Cohesiveness	0.0278	0.7418	0.0278	0.4016	/	/	0.0278	0.3701	/	/	/	/	/	/
	Springiness	1.8061	0.6656	1.8061	0.9040	/	/	1.4547	0.0457	/	/	/	/	/	/
	Gumminess	341.24	0.3364	341.24	0.7789	/	/	241.3	0.1201	/	/	/	/	/	/
	Chewiness	296.92	0.3891	296.92	0.7663	/	/	213.38	0.1221	/	/	/	/	/	/

Note: SEM—Standard error of means; Fat*Lean: interaction between Fat and lean; Fat*S: interaction between Fat and storage; Lean*S: interaction between Lean and storage; Fat*Lean*S: interaction between Fat, lean and storage; Data from three independent trials were analyzed using PROC MIXED procedure. Main effect of fat and lean types represent fat and lean from either in utero heat stressed or thermoneutral pigs as well as their interactions on the tested parameters were evaluated. SEM and *p* values were presented; Main factor with a *p* value < 0.05 is considered as having significant impact on the measured attributes, and detailed comparison will be performed and listed in the corresponding tables.

**Table 3:** Proximate composition of pork patties and emulsion sausages.

		Moisture %	Protein %	Ash %	Fat %
	TN_0	69.09 ^A^	23.80 ^A^	1.32 ^A^	5.79 ^C^
Patties	HS_0	69.39 ^A^	24.19 ^A^	1.33 ^A^	5.08 ^C^
	TN_25	53.90 ^B^	19.15 ^B^	1.03 ^B^	25.93 ^A^
	HS_25	55.38 ^B^	19.33 ^B^	1.05 ^B^	24.24 ^B^
	HSF–HSL	57.10 ^b^	12.25	2.87	27.78 ^a^
Emulsion sausages	HSF–TNL	58.79 ^a^	12.82	2.99	25.40 ^b^
	TNF–HSL	58.26 ^ab^	13.15	2.86	25.72 ^b^
	TNF–TNL	57.81 ^ab^	13.10	2.97	26.12 ^b^

Note: HS_0, HS_25, TN_0, and TN_25 represent pork patties formulated with 0% of pork back fat, 25% of IUHS pork back fat + 75% of IUHS lean meat, 0% of IUTN back fat and 25% of IUTN back fat + 75% of IUTN lean meat, respectively; HSL–HSF: In utero heat stress (IUHS) Lean + IUHS Fat, HSF–TNL: IUHS Fat and in utero thermoneutral (IUTN) Lean, TNF–HSL: IUTN Fat and IUHS Lean, TNL–TNF: IUTN Lean and IUTN fat; Different letters (A–C) in the same column indicate significant difference (*p* < 0.05) between samples for patties; Different letters (a,b) in the same column indicate significant difference (*p* < 0.05) between samples for emulsion sausages.

**Table 4:** Textural profile analysis, cooking loss and fat-binding capacity of pork sausage emulsions.

Pork Emulsion	Hrds (g)	Adhes (g*sec)	Resi (%)	Cohes (%)	Spris (%)	Gums (kg)	Chws (N)	CL (%)	FBC (%)	pH
HSF–HSL	5197	−20.08	34.86	0.67	78.67	3498	2763	0.74	6.08	5.68 ^b^
HSF–TNL	5569	−18.64	36.33	0.69	81.02	3557	3103	0.56	5.71	5.85 ^a^
TNF–HSL	5889	−17.70	35.45	0.67	81.40	3619	3220	0.89	5.65	5.90 ^a^
TNF–TNL	5162	−23.25	36.04	0.69	78.98	3571	2826	0.76	5.11	5.93 ^a^

Note: Hrds, Adhes, Resi, Cohes, Spris, Gums, Chws, CL, and FBC mean Hardness, Adhesiveness, Resilience, Cohesiveness, Springiness, Gumminess, Chewiness, Cooking Loss, and Fat-Binding Capacity, respectively. HSL–HSF: IUHS Lean + IUHS Fat, HSF–TNL: IUHS Fat and IUTN lean, TNF–HSL: IUTN Fat and IUHS lean, TNL–TNF: IUTN Lean and IUTN fat. A total of three batches was conducted, with three replications for each treatment in each batch (*n* = 3), measurements for TPA were performed in duplicate, and CL and FBC were performed in triplicate. Different letters (a,b) in the same column indicates significant difference (*p* < 0.05).

**Table 5:** Textural profile analysis of pork patties.

Treatment	Hrds (g)	Adhes (g*sec)	Resi (%)	Cohes (%)	Spris (%)	Gums (kg)	Chws (N)
HS_0	18,119 ^a^	0.20	29.57	0.68	82.43	12,265	10,124
HS_25	4897 ^c^	0.11	17.85	0.46	62.61	2263	1444
TN_0	15,151 ^b^	0.11	26.77	0.65	81.11	9790	7952
TN_25	6355 ^c^	0.11	17.78	0.45	65.84	2942	2015

Note: Hrds, Adhes, Resi, Cohes, Spris, Gums, and Chws mean Hardness, Adhesiveness, Resilience, Cohesiveness, Springiness, Gumminess, and Chewiness, respectively. HS_0, HS_25, TN_0, and TN_25 represent pork patties formulated with 0% of pork back fat, 25% of IUHS pork back fat + 75% of IUHS lean meat, 0% of IUTN back fat, and 25% of IUTN back fat + 75% of IUTN lean meat, respectively. A total of three batches was conducted, with three replications for each treatment in each batch (*n* = 3), and measurements for TPA were performed in duplicate. Different letters (a–c) in the same column indicates significant difference (*p* < 0.05).

**Table 6:** Color attributes of pork patties.

		L*	a*	b*	Hue	Chroma
In utero condition (IUC)	IUHS	54.76	13.85 ^a^	7.48	28.5 ^b^	15.93 ^a^
	IUTN	55.44	12.27 ^b^	7.57	31.20 ^a^	14.50 ^b^
Fat content (FC, %)	0	51.14 ^b^	11.46 ^b^	5.23 ^b^	25.54 ^b^	12.77 ^b^
	25	59.07 ^a^	14.66 ^a^	9.52 ^a^	34.24 ^a^	17.66 ^a^
IUC–FC interaction	HS_0	50.11 ^c^	11.77 ^bc^	4.96 ^d^	22.63 ^c^	12.80 ^c^
	HS_25	59.41 ^a^	15.93 ^a^	10.00 ^a^	34.52 ^a^	19.07 ^a^
	TN_0	52.17 ^b^	11.15 ^c^	6.09 ^c^	28.45 ^b^	12.74 ^c^
	TN_25	58.72 ^a^	13.40 ^b^	9.04 ^b^	33.96 ^a^	16.25 ^b^
Storage (d)	0	56.97 ^a^	14.48 ^a^	8.18 ^a^	28.91 ^b^	16.73 ^a^
	1	56.97 ^b^	14.30 ^a^	7.54 ^abc^	28.70 ^b^	16.38 ^ab^
	2	54.68 ^b^	12.41 ^ab^	7.15 ^bc^	29.05 ^b^	14.41 ^bc^
	3	54.54 ^b^	12.00 ^b^	7.72 ^ab^	31.95 ^a^	14.35 ^c^
	4	54.98 ^b^	12.12 ^b^	7.02 ^c^	30.84 ^ab^	14.21 ^c^

Note: IUHS and IUTN represent in utero heat stress and in utero thermoneutral, respectively. Different letters (a–c) in the same column indicate significant difference (*p* < 0.05).

**Table 7:** Fatty acid profiles of subcutaneous fat collected from either in utero heat stress (IUHS) or in utero thermoneutral (IUTN) pigs.

Fatty Acids	IUHS	IUTN	*p* Value	SEM
C8:0	0.01	0.01	0.32	0.00
C10:0	0.07	0.08	0.13	0.01
C12:0	0.07	0.08	0.32	0.01
C14:0	1.28	1.35	0.22	0.08
C14:1 cis9	0.01	0.01	0.18	0.00
C15:0	0.05	0.05	0.59	0.01
C16:0	25.93	26.54	0.51	1.11
C16:0 15-methyl	1.13	1.04	0.35	0.12
C16:1 cis9	1.94	1.90	0.75	0.14
C16:0 14-methyl	0.22	0.15	0.14	0.03
C16:1 cis7	0.01	0.00	0.21	0.01
C17:0	0.37	0.32	0.50	0.10
C17:1 cis10	0.22	0.19	0.27	0.05
C18:0	13.45	14.03	0.68	1.27
C18:1 trans11	0.15	0.15	0.70	0.02
C18:1 cis9	30.22	30.07	0.90	0.97
C18:1 cis11	2.67	2.53	0.39	0.15
C18:1 cis13	0.02	0.01	0.33	0.01
C18:2 cis9 cis12	19.32	18.96	0.77	1.87
C19:0	0.04	0.04	0.93	0.01
C19:1 cis10	0.023	0.02	0.93	0.00
C19:1 cis UN	0.05	0.04	0.17	0.01
C20:0	0.21	0.21	0.72	0.02
C18:3 cis9 cis12 cis15	0.45	0.46	0.87	0.06
C20:1 cis11	1.00 ^a^	0.79 ^b^	0.01	0.05
C20:2 cis11 cis14	0.77	0.60	0.08	0.06
C20:3 cis5 cis8 cis11	0.02 ^b^	0.03 ^a^	0.05	0.00
C20:3 UN	0.01	0.02	0.30	0.00
C20:3 cis8 cis11 cis14	0.08	0.09	0.38	0.01
C22:0	0.00	0.00	0.91	0.00
C20:4 cis5 cis8 cis11 cis14	0.14	0.14	0.77	0.01
C22:4 cis7 cis10 cis13 cis16	0.08	0.09	0.38	0.01
SFA	41.50	42.71	0.64	2.36
BCFA	1.34	1.19	0.26	0.15
MUFA	36.30	35.73	0.66	1.20
PUFA	20.86	20.37	0.72	2.01
n3PUFA	0.45	0.46	0.87	0.06
n6PUFA	20.39	19.87	0.70	1.94
n6/n3	45.52	44.18	0.50	2.13
LCPUFA	1.10	0.96	0.21	0.09
LCn3PUFA	0.00	0.00	/	/
LCn6PUFA	1.07	0.92	0.16	0.08
Trans FA	0.15	0.15	0.70	0.02
CLA	0.00	0.00	/	/
SI	0.73	0.77	0.63	0.08

Note: SFAs, BCFAs, MUFAs, PUFAs, LCPUFA, CLA, and SI represent saturated fatty acids, branch-chained fatty acids, mono-unsaturated fatty acids, polyunsaturated fatty acids, long-chain polyunsaturated fatty acids, conjugated linoleic acids, and saturation index, respectively. SEM: Standard error of means; different letters among results in the same column are significantly different from each other at the confidence level of 95% (*p* < 0.05).

## Data Availability

Data are available on request to the corresponding author.
